# Etiology of acute diarrhea in patients requiring hospitalization in Clinical Infectious Diseases Hospital - Constanța

**DOI:** 10.1186/1471-2334-14-S7-O27

**Published:** 2014-10-15

**Authors:** Anca Dumitrescu, Sorina Carp, Maria Margareta Ilie, Elena Dumea, Sorin Rugină, Stela Halichidis, Simona Claudia Cambrea

**Affiliations:** 1 Clinical Infectious Diseases Hospital of Constanța, Romania; 2 Faculty of Medicine, “Ovidius” University, Constanța, Romania

## Background

Acute diarrhea (AD) plays an important role in human pathology because of its high incidence, especially in children. The study aims to evaluate the etiology of AD and bacterial isolated drug resistance for hospitalized patients during July 2013 and June 2014.

## Methods

During July 2013 and June 2014 stool cultures for the identification of the bacterial pathogen agents were performed and we used latex agglutination tests for the detection of rotavirus for patients who presented at the Infectious Diseases Hospital with AD.

## Results

In the study period 3,929 patients with AD (with 353 positive cases) presented to the Infectious Diseases Hospital out of which 2,550 children (with 301 positive cases) and 1,371 adults (with 52 positive cases). In children rotavirus enteritis ranked first in the etiology of infectious enteritis with a confirmed etiology of 62% over the entire period (186 cases) with a peak incidence in January-March – 78% of enteritis with infectious etiology confirmed (92 cases). Of bacterial causes of enteritis in children under 2 years, *Klebsiella* ranked first with 60% (32 cases), followed by enteropathogenic *E. coli*, with 25.9% (14 cases). The isolated strains of *Klebsiella* were 100% sensitive to imipenem, 92% sensitive to quinolones, 80% sensitive to ceftriaxone but only 33.3% sensitive to amoxicillin/clavulanic acid. In children over 2 years, the most frequently encountered bacteria was *Salmonella* spp. – 47.5% of bacterial enteritis (29 cases), followed by *Shigella* spp. – 26.2% (16 cases) and *Campylobacter* – 11.47% (7 cases).

The most common cause of enteritis with confirmed infectious etiology in adults was *Salmonella* spp., with a percentage of 47.9% (23 cases), followed by *Clostridium* spp. with 29.1% (14 cases) and *Shigella* spp. with 16.6% (8 cases). The strains of *Salmonella* spp. that had been isolated from adults were 100% sensitive to: quinolones, cephalosporins, imipenem, gentamicin, but 26% (6 cases) were resistant to tetracycline.

## Conclusion

Rotavirus is the most common cause of hospitalized enteritis in children. Both in children over 2 years and adults the most common pathogen in bacterial enteritis is *Salmonella*. A great percentage of post-antibiotic AD in adults is caused by *Clostridium* spp.

